# P57 Improving access to education and engagement on antimicrobial resistance for students from diverse backgrounds

**DOI:** 10.1093/jacamr/dlaf118.064

**Published:** 2025-07-14

**Authors:** Josephine Owen, Oliver Burley, Katarina Johnson, Zeshan Riaz, Bamidele Famokunwa

**Affiliations:** Medical Affairs, Pfizer Ltd, Tadworth, UK; Medical Affairs, Pfizer Ltd, Tadworth, UK; Medical Affairs, Pfizer Ltd, Tadworth, UK; Medical Affairs, Pfizer Ltd, Tadworth, UK; Medical Affairs, Pfizer Ltd, Tadworth, UK

## Abstract

**Background:**

In 2019, antimicrobial resistance (AMR) was linked to 35 200 deaths in the UK.^1^ To raise awareness of this growing issue, in 2023 we held a ‘Superbugs: Join the Fight’ 3 day event in London, for students aged 7–14, focussing on education around infection control, AMR and how science, technology, engineering, and mathematics (STEM) careers are addressing the challenge of AMR. The 2023–24 ESPAUR report highlights that the impact of AMR is unequally distributed across the country, emphasizing the association between areas of deprivation and higher AMR prevalence, in areas like the North-West of England.^2^ As a result, we took the ‘Superbugs: Join the Fight’ event to Manchester, an area with higher levels of AMR.

**Objectives:**

To improve students’ knowledge around AMR, infection control, and how antimicrobial stewardship (AMS) can be implemented. To demonstrate that the teaching methodology used at the London event, is effective and accessible for a wide range of ages, sexes, and neurodiversities. To help facilitate equality in education by providing free education opportunities.

**Methods:**

‘Superbugs: Join the Fight’ Manchester was a 1 day event consisting of 2 sessions each with 2 rotations. The first rotation was an interactive session focused on the spread of superbugs and the reduction of AMR, involving a coughing simulator and barrier protection methods. The second focused on careers in STEM led by 2 AMS pharmacists, involving a Q&A session with STEM ambassadors, and videos sharing the stories of different scientific careers. This was the same teaching methodology used previously in London. Before and after each session students self-evaluated their level of AMR knowledge as either ‘nothing’, ‘a little’ or ‘a lot’. Stickers were also given out to reward engagement. Following the session, students were asked to share feedback and key learnings from the workshops. When sending invites to schools the focus was on secondary education (students aged 11-16), special educational needs and disability (SEND) schools, and those with high percentages of students eligible for free school meals (FSM), an indicator of deprivation.^3^

**Results:**

Over 130 students attended the event from 5 different schools. 74% of those students were female, around 90% of the students were eligible for FSM, and 2/5 schools were SEND. Across both sessions and consistent with findings from ‘Superbugs: Join the Fight’ London, we saw a 92% increase in AMR knowledge pre- and post-workshop based on student's self-evaluation, and in total 840 stickers were given out for engagement.

**Conclusions:**

The reported knowledge increases achieved by each event demonstrates that the teaching methodology employed is effective and accessible for students from a range of backgrounds, ages, and neurodiversities. Raising awareness of AMR and inspiring antibiotic guardians of the future is vital to addressing AMR. This initiative should be expanded to other geographical areas, to help raise awareness across the UK and engage future generations.
